# Reaching national Covid-19 vaccination targets whilst decreasing inequalities in vaccine uptake: Public health teams' challenges in supporting disadvantaged populations^☆^

**DOI:** 10.1016/j.puhip.2024.100551

**Published:** 2024-10-25

**Authors:** J. Lecouturier, Michael P. Kelly, Falko F. Sniehotta

**Affiliations:** a Population Health Sciences Institute, Newcastle University, Newcastle, UK; b Department of Public Health and Primary Care, University of Cambridge, Cambridge, UK

**Keywords:** Public health, Local authority, COVID vaccination, Health inequalities, Qualitative research, Community assets

## Abstract

**Background:**

UK local authority public health teams (LAPHT) supported delivery of the Covid-19 vaccination programme, particularly to disadvantaged populations. We explored the challenges encountered and lessons learnt by LAPHTs in tackling low Covid-19 vaccine uptake. The aim of this study was to understand what works, and how, in addressing local inequalities in relation to uptake of the Covid-19 vaccination with a view to generalising insights to building back fairer after the pandemic and into the future.

**Study design:**

Qualitative.

**Method:**

We conducted in-depth on-line interviews with Directors of Public Health or their representatives from 21 English local authorities covering a total population of over 8 million people. Data were analysed thematically.

**Results:**

Accessing the requisite (and accurate) data, engaging with communities, and working with National Health Service (NHS) organisations presented challenges in delivering initiatives to improve vaccine uptake, particularly for disadvantaged groups. LAPHT's assets beneficial to the programme - in-depth knowledge and experience of their communities and locality - were not considered in the national vaccination programme. Community engagement and relationships with local NHS featured heavily in the majority of LAPHTs responses to improving vaccine uptake rates.

**Conclusions:**

Incorporating local public health infrastructure, expertise and existing relationships into national vaccination planning during epidemics or pandemics is crucial. Community engagement and good relationships with NHS staff help to reach and serve disadvantaged populations. How these can be developed and maintained in the longer term without future investment was a concern. Future research should explore the design and implementation of PH and NHS joint service delivery models to tackle health inequalities, informed by experiences of the Covid-19 vaccination programme and with input from community partners.

## 1 Introduction

In England in 2013 public health was relocated from the National Health Service (NHS) to local authorities (LAs) [1]. Directors of Public Health (DsPH) are responsible for health improvement, health protection and health care service planning and commissioning. DsPH ‘must be empowered to have oversight and influence’ across these domains ‘within local authorities, the NHS and primary care, and other sectors and agencies to secure the improving health of their population and its protection’ [2]. UK local authority public health teams (LAPHT) had a crucial role to play during the Covid-19 pandemic. The Covid-19 vaccination programme, launched in December 2020, was delivered by the NHS; LAPHTs worked alongside to promote the programme. LAs were awarded Contain Outbreak Management Funding by central government, the amount based upon population size and level of deprivation [3]. Community Covid Champion funding to boost vaccination uptake was also available on application from central government [4].

The initial approach was to deliver the vaccine sequentially to nine cohorts based on nationally set criteria [5]. In the UK, as early as May 2020, it emerged that ethnic groups were disproportionately affected by the virus, with higher hospital mortality rates [6]. This was attributed to higher rates of co-morbidities, deprivation, racism, discrimination, and greater exposure to Covid-19 through occupation [7]. Once a vaccine was available there were calls to prioritise minority ethnic groups [8] though uptake in these groups remained low [9].

In the UK the Covid-19 vaccination was offered through mass administration sites (e.g. football stadia), hospitals, general practices and community pharmacies [10]. At this time there was little published literature on best practice in supporting disadvantaged groups with Covid-19 vaccination. In previous research regarding pandemic influenza immunisation, a review identified 12 strategies to improve uptake, though the majority of these did not consider the needs of disadvantaged communities [11]. The strategies address the individual (partnering with community leaders, campaigns, outreach clinics), provider (additional staff to immunise) and structural levels (prioritisation of groups for immunisation, communications developed with community partners and programmes tailored, and responsive, to the needs of the population). More recent research with vulnerable populations (migrants, refugees and homeless) report non-standard modes of delivering influenza immunisation, namely mobile outreach clinics, have improved uptake [12].

In the midst of the Covid-19 pandemic we conducted a qualitative study to explore LAPHT experiences in strategic planning and implementing initiatives to maximise vaccination uptake, particularly in disadvantaged groups, the challenges and lessons learnt. The aim was to gain insights for building back fairer after the pandemic and into the future.

## 2 Methods

This was a purposive sample to represent the different LA structures, populations and geography. DsPH and/or other LA staff supporting the vaccination programme were the target population. Study details were circulated through the Association of Directors of Public Health, the National Institute for Health and Care Research Clinical Research Network and the Faculty of Public Health. As recruitment progressed a matrix was created with local authority structure, geographical area, population size, and ethnic diversity; this enabled the team to identify gaps in the sample and target specific local authorities. Data were collected from July 2021 to March 2022.

Qualitative in-depth interviews were conducted on-line by an experienced female qualitative researcher (JL) using a topic guide (supplementary document) developed and tested with the wider research team. To make best use of the time agreed for the interview, consent forms were sent by email a few days beforehand. At the interview, interviewees were asked if they agreed to the consent form statements: this and the interview were digitally recorded. Field notes were made throughout the interviews. Soundfiles were transcribed and anonymised. Transcripts were uploaded to NVivo (14) and analysed using an inductive thematic approach (Table 1) [13]. The coding frame (Supplementary file) was agreed by all authors. Data were coded by one researcher (JL).

**Table 1:** Steps in thematic analysis.

**Step 1: Familiarisation with data** – reading, re-reading and listening to recordings of interviews/focus groups
**Step 2: Generate initial codes –** systematically record features of the data that are interesting across the data
**Step 3: Identify themes –** coded extracts are sorted into overarching themes. Subthemes are developed where appropriate
**Step 4: Review of themes –** at this stage themes are combined, refined, redefined or separated. From this map or framework is devised
**Step 5: Defining and naming themes –** another stage of refinement of the themes and sub-themes and the addition of concise working definitions of each theme

## 3 Results

DsPH from 25 LAPHT responded via email and expressed an interest in the study. As four were too busy to participate, 28 people (sometimes a second person was included to obtain the full picture) were interviewed from 21 English LAs. These LAs represented a population of 8+ million people, had a mix of structures (two tier: county council 3; unitary areas: unitary authorities 12, Metropolitan Borough Councils 3; London Boroughs 3), and rural/urban/coastal areas (Table 2). Most had areas of high deprivation and varying proportions of people from ethnic minorities [14].

**Table 2:** Local authority area classification (coastal).

Urban with city and town	Urban with significant rural	Rural	Urban with minor conurbation	Urban with major conurbation
8 (3)	4 (4)	2	3 (1)	4

Interviewees were primarily DsPH (11), a mix of public health consultants, registrars and practitioners (10), and other Covid-19/vaccination leads (7). The majority had been employed in a public health capacity for over five years and ranged from 18 months to 35 years. Interviews lasted 54 min on average (range 39–69 min). Data saturation was achieved when no new themes were being identified.

### 3.1 Challenges to implementing initiatives

LAPHTs faced numerous barriers to increasing vaccination uptake: Covid-19/vaccination misinformation (at home and abroad); the anti-vax faction; and lack of public trust in the government and vaccines. LAPHTs had tried initiatives/activities (Supplementary documents), sometimes delivered alongside NHS partners, which targeted the Three C's of confidence, convenience, and complacency [15] to improve uptake in disadvantaged groups.

**Fig. 1:**
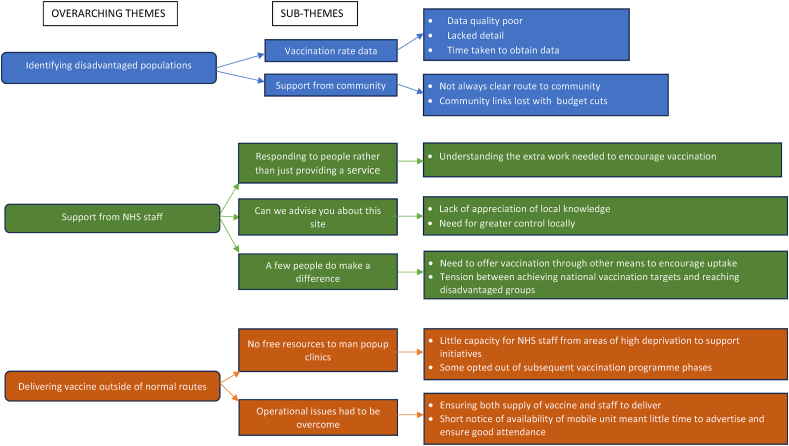
Themes relating to the challenges of implementing initiatives to increase vaccination uptake in disadvantaged groups.

There were three overarching themes (described below with subthemes) of the challenges they experienced: identifying disadvantaged populations; gaining support from NHS staff; and delivering the vaccine outside of the normal route (Fig. 1).

#### 3.1.1 Identifying disadvantaged populations

##### 3.1.1.1 ‘Without the granular data you can't target your vaccination programme’

LAPHTs required both vaccine uptake and detailed population data to identify *where* to target initiatives. Interviewees reported the decision as to which data were available to DsPH, was made centrally without local consultation. Four sources of national vaccination rate data (Table 3) were available on application for DsPH and a few nominated staff. Most found the data inadequate in quality, level of detail and time taken to obtain. Data access improved over time, though in January 2022 one interviewee said ‘we can't tell ethnicity so it's still a bit limited but good for geographical inequalities' (Site18). Frequently public health intelligence staff patched data together from all sources, aligning this with local NHS vaccination rate data when available.

**Table 3:** Data sources on Covid-19 vaccination available (at March 2022) to public health teams.

• NHS Foundry - LSOA (lower layer super output areas^a^)
• Public Health England - weekly data o Lower tier^c^ local authority, detailed ethnic group (17 categories) and gender oMSOA (middle layer super output areas^b^), higher level ethnic group (6 categories)
• Heat maps from Clinical Commissioning Group's performance team on number vaccinated
• NIMS (National Immunisation Management System) oDaily - age and lower tier local authority, first and second doses – uses Foundry data oWeekly – For local authority middle tier^d^ is by age for each dose (NIMS only) oMonthly – NHS region, age, ethnicity, gender (also from PHE/UKHSA data)

a areas with an average of approximately 1500 residents or 650 households.

b areas with an average population of 7500 residents or 4000 households.

c lower tier - District, Borough or City Council.

d upper tier - County or Shire Council.

Inaccuracies in national data affected targeting initiatives and understanding uptake rates. The latter could reflect badly on an LAs performance or overestimate numbers vaccinated. In the data providers' defence, one interviewee highlighted the difficulties collating rapidly changing vaccination rate data and providing it ‘in a form that didn't compromise information governance’ (Site5). The names and addresses and numbers who had *contracted* Covid was eventually available to LAPHTs, but not for those unvaccinated:

‘One criticism of the vaccination programme was they should have provided DsPH with access to the individual line list in the same way they had for cases and contacts. That would have made it much more granular in terms of our understanding.’ DPH/Site2/Aug21

General practices and Clinical Commissioning Groups also held the required data, but the sharing landscape was complex, and problematic when formal agreements were not in place. These data also had deficiencies; ethnicity and learning disabilities were often not recorded, or ethnicity recorded in a generic way that was unhelpful in identifying specific populations.

##### 3.1.1.2 ‘It's just so critical having good, well respected and trusted voices within the local community’

Interviewees considered a “bottom-up” approach crucial to tackling inequalities. Links between LA and communities were considered key to facilitating engagement in the vaccination programme. Yet engaging with communities was not easy or even possible in LAs where there was no clear route in or identified faith/group/community leader. For financial reasons some LAs were unable to maintain community links. Due to national budget cuts, one LA lost the engagement teams they believed were so crucial in establishing community links. Without this resource LAPHT had to try to rebuild specifically in response to the pandemic; where they had been retained this was invaluable.

‘The reason we’ve been able to work the way we have is there’s been consistent investment in community development, [, , ,] that fell by the wayside in a lot of areas [LA] has maintained. They really worked hard to maintain the relationships over the years and what we’re bearing up now is the fruit of those relationships.’ Site7a/Sep21

LAs with a recent sharp increase in residents from minority ethnic groups also struggled to engage, as the groups were geographically dispersed with no established church/community centre/group. Interviewees found it difficult to connect with populations from Eastern Europe, targeting workplaces, shops, cafes, and schools with little success. These shops and cafes were often not frequented by Eastern Europeans. It was argued that LAs need investment to develop and maintain community engagement.

#### 3.1.2 Gaining support from NHS staff

##### 3.1.2.1 ‘Responding to people rather than just providing a service’

Interviewees unanimously praised the vaccination programme staff. However, several interviewees experienced local NHS staffs’ hesitancy to engage in initiatives that went beyond the usual vaccination route, though this did change over time. Because of the vaccination programme scale and pace, there were pressures on NHS staff and concerns about wasting resources (vaccines and staff). Nevertheless, interviewees believed there were additional factors contributing to their caution. The first was said to be the NHS mindset of believing it sufficient to *offer* the vaccine.

‘That’s a very NHS thing you know “We tell you when to come and you come and we’ll give it to you” and that’s not how it works for people. I think – surprise, surprise – quite quickly those doing the vaccine discovered actually after-work and the weekends and making it available as a walk-in works better.’ Site12/Dec21

NHS staff did not always consider the extra work required to support and encourage the public to be vaccinated. Some NHS staff were surprised when they encountered those who had not been vaccinated as soon as the opportunity arose and did not appreciate the many reasons why people may act in this way. It was thought the NHS had not picked up the baton and carried on the work to ensure vaccination uptake, particularly in disadvantaged groups, when public health moved from the NHS to LAs. Interviewees believed inequalities should be high on the NHS and public health agenda; a few said although on the NHS agenda, it took time for them to act upon this. However, it was recognised that NHS staff attitudes changed over time.

‘I have to say this has been one of the most interesting periods for doing what we’ve talked about for 30 years in the NHS which was actually responding to people as opposed to just providing services.’ Site7b/Sep21

##### 3.1.2.2 ‘Can we please advise you about this site?‘

Another factor contributing to local NHS hesitation in adopting different initiatives, was a lack of appreciation both of LAPHTs role and knowledge of their communities/local area. Again, this was attributed to public health's move to LAs, where the NHS institutional memory of the DsPH role and specific expertise had been forgotten. In certain cases, NHS staff unfamiliar with the local area did not seek the LAPHT input and help. Some had to convince NHS staff to invest in different initiatives and this took time:

‘That was the challenge at the start. We were constantly being asked to prove it or go and engage and come back with evidence, rather than us just going on into these communities. I think that’s why it took three months.’ Site16/Jan22

As partnerships between NHS and LAPHT developed so did trust. In some cases, NHS staff relinquished control and LAPHT took the lead. As one interviewee stated, ‘It was a big risk for the NHS because they don't normally like to let go of things, but it worked, it absolutely worked’ (Site7b). When NHS partners engaged, they saw the value of delivering the vaccination outside the usual settings.

‘It was actually a much longer conversation over quite a few months, and them going, “Oh alright then”, and then, “Oh wow, look at the people you get when you just park a bus in a supermarket car park. Amazing. Completely different people than the people we get at the mass vaccination centre”, and all credit to them because they really took it on board and have really run with it.’ Site18/ Jan22

With greater local control LAPHTs were able to respond more efficiently. In one LA, offering vaccination drop-in clinics for those who were not registered with a GP ‘changed the picture dramatically’ (Site10). Regarding central government, throughout the pandemic DsPH had pushed back about the restrictions on LAPHTs and stressed the need for greater local control. As time progressed the strategy was relaxed and, echoing others' views, one interviewee reported ‘things improved when the brakes were taken off’ (Site10).

‘NHS England planning for the Booster Programme, did give local areas quite a bit of flexibility about how they deliver it, which was good. It was less top/down command and control than the earlier phases of the programme.’ Site1/Jul21

##### 3.1.2.3 ‘A few people do make a difference’

Another challenge to LAPHT was the standard vaccinations settings and focus on vaccination rates. Interviewees thought these increased inequalities, put undue pressure on LAPHTs and allowed them little flexibility to respond to their local situation.

‘It’s NHS command and control at its best. Never mind the quality, get the numbers through which works well if you’re affluent, white and got a car and time. It doesn’t work for my communities.’ Site12/Dec21

Vaccinating one person from a disadvantaged group was of high value to interviewees and a potential route to access others. However, there was an obvious tension between this, and the associated NHS staff costs mobilised to deliver the vaccination outside the usual setting often in much smaller numbers. One interviewee noted that Primary Care Networks^1^ (PCNs) are businesses and unlikely to deliver the vaccine if cost exceeded reimbursement. LAPHT could not guarantee attendance and were concerned about wasting resources.

‘We’re constantly asked by our chief exec why are we not going into these communities to do this percent of people and it’s because of the amount of wastage. It’s not ethical when you think if we were to keep vials back at the vaccination centre we could vaccinate 20 people, whereas we might only vaccinate five somewhere else. It’s been a really difficult decision.’ Site09/Oct21

#### 3.1.3 Delivering the vaccine outside of the normal route

##### 3.1.3.1 ‘No free resource to staff popups’

LAPHTs were reliant on NHS staff to engage in initiatives but there were capacity issues. PCNs/general practices in areas of high deprivation and/or a larger proportion of disadvantaged groups had to commit more resources to encourage vaccine uptake. They had little capacity to support LAPHT initiatives, which impacted on the ability to offer more flexible vaccination opportunities.

Unlike in Phase 1, Phase 2 and the booster programme, PCNs could opt-out of subsequent vaccination programme phases. Difficulties securing staff to deliver the vaccine through the LAPHT initiatives was compounded in opt out areas.

‘We went from two PCNs to only one PCN delivering it across the whole area because it wasn’t viable for them to do it and they were getting back to business as usual […] because of the pressure on GPs … We are in a very difficult position where some of the NHS providers are jumping ship. We have no control over that.’ Site9/Oct21

##### 3.1.3.2 ‘Operational issues had to be overcome’

Vaccination outside the standard settings was challenging logistically. As one interviewee described, these initiatives involved the organisation of two halves; one half the LAPHT could manage. and the NHS delivery half over which they had little control. There were concerns about beginning community engagement work with uncertainties over vaccine supply and ability to offer the initiative.

‘It’s not just a case of wanting to do something, you need to have the vaccinators and the vans or the mobility and the vaccine supply and all of those obviously need to come together and some ideas didn’t take off fairly early on because we couldn’t get all of those things together.’ Site5/Sep21

LAPHT concerns about potential low numbers meant promoting and advertising the event was most important. Several interviewees had relatively short notice in which to publicise a mobile unit/pop-up clinic on a particular day.

The lessons learnt in implementing initiatives are presented in Table 4.

**Table 4:** Lessons learnt from delivering the initiatives to improve vaccination uptake.

The importance of building and maintaining relationships with NHS colleagues and with the community					
	Why Important?	Impact of working with NHS/communities	Comments	Concerns	Comments
**NHS Colleagues**	• NHS impact on the implementation of the initiatives to reach disadvantaged groups • Potential for joint NHS/PH working to address other health issues.	• Improved relations between the NHS and public health/LA • Existing barriers were overcome, and people had stopped working in silos.	*‘It's pulled a lot organisations far closer together, which I think in the longer term is going to be a real positive lesson learned that we can be far more successful if we do all of this together rather than just trying to tackle it all as individual agencies.’ Site 1 – July 2021*	NHS and public health will return to silo working	*‘What we really don't want to lose is what we've got now for future vaccinations and other types of programmes that we deliver jointly with the NHS.’ Site 19 – March 2022*
**Community engagement**	• Provided an understanding of communities at the grassroots level • Beneficial to engage communities to address other public health issues	• Played a key role, providing both intelligence on the current issues of concern for local groups and a route to those who may not engage with the LA and NHS. • Instrumental in a number of initiatives to improve vaccination uptake	*‘I think at a community level we, like many councils, had disinvested in community development, the relationships weren't there. We rebuilt that massively. … One of the chairmen of the mosques … said this is the first time in his life that the mosques in the city have acted together and … we must not let it go. And so I think that's a really important one, all the lessons that we've learned about engagement, about comms, we must not walk away from them.’ Site 4 – August 2021*	Lack of resources to maintain community partnerships	

## 4 Discussion

This study highlighted the crucial role of LAPHTs in the Covid-19 vaccination programme in England, supporting people through the standard vaccination routes and providing alternative options. The latter was particularly important for disadvantaged groups. Their main challenges to implementing initiatives to improve vaccine uptake related to local NHS staff and central government.

Everyone remarked upon NHS colleagues' dedication and extraordinary work, particularly the rapid progression through the vaccination programme cohorts. However, in implementing initiatives, LAPHTs encountered challenges with NHS staff vaccinators' capacity, tensions between achieving a critical mass of vaccinations and investing time and effort to reach the most vulnerable and disadvantaged, and in convincing NHS staff to engage. Relationships with local NHS improved over time, something LAPHTs hoped would continue in the longer term. Regarding central government, most LAPHTs had difficulties accessing the requisite vaccination rate data which significantly affected identifying whom to target with the initiatives. Local/national NHS and central government's lack of recognition of the DsPH role, skills, knowledge, and experience underpinned many issues. DsPH were not involved in Covid-19 planning and strategy development, and initially were unable to work flexibly to respond to local need. Though interviewees believed DsPH are best situated in LAs, some thought their specific expertise, knowledge, and importance as part of a multi-agency pandemic response, had been forgotten. This suggests policy surrounding the original public health move in 2013 lacked clarity about NHS and public health roles and responsibilities.

Routes into disadvantaged communities provided opportunities to support people with other health issues and the potential to build trust with health providers. These relationships held clear benefits for the future of public health if they could be maintained. Community engagement was easier where links had survived spending cuts, and for those successful in obtaining Covid-19 funding from central government. The latter enabled sufficient but short-term community engagement and LAPHTs were concerned how this could be sustained or developed in the longer term without extra resources. Some LAPHTs had used this funding to recruit staff to support the vaccination programme and not all contracts could be extended beyond the pandemic. The downside was the loss of knowledge gained about engaging with disadvantaged communities. Drawing upon the help of others within the LA was not a long-term option for many and the need for adequate funding to support vaccination programmes and future public health initiatives was raised.

A number of reports have focused on *pandemic* response experiences [[16], [17], [18], [19]]. Three key messages from a UK report of DsPH experiences from 2020 until May 2021, five months into the vaccination programme [16], resonate with the current study findings. First, central government's lack of engagement with DsPH on the national testing strategy, second, the importance of DsPH knowledge and expertise in addressing health inequalities and third, historical underinvestment in LAs/public health. Outside the UK, although governmental structures and public health systems differ, others have reported tensions in the Covid-19 response between local and national structures [17], public health workforce shortages and long-term underinvestment [18]. On a more positive note, the pandemic raised awareness of public health with the public and across health systems and brought public health professionals in closer proximity to key decision-makers [19]. As in the current study, it was hoped that health provider and public health collaborations developed during the pandemic, and extra funding, would be continued in the longer term to support public health teams to address the needs of the population [18].

In recent years in the UK there has been a fall in childhood vaccination rates [20] and there are inequities for minority ethnic groups with lower vaccination rates [21]. The resurgence of measle outbreaks is a major concern in the UK [22] and globally [23] with a rise in the number of cases. In the UK the fall in MMR vaccination rates has been attributed to access issues and parental attitudes to vaccination [24]. Offering vaccination in different settings and at different times and engaging with communities have been suggested as ways of tackling barriers to uptake [25] and, as our work has demonstrated, LAPHTs could play a key role in this.

### 4.1 Limitations of this study

Data were collected over a difficult and pressured time for DsPH. Four of the 25 LAPHT who initially expressed an interest did not proceed to interview. There were two geographical areas from our matrix we wished to include but had no response from the DsPH. We acknowledge that we were unable to capture the views of all of our intended LAPHTs. However, many of the LAPHT interviewees were in close contact with, or part of a network of, Covid-19 response public health teams, and reported that all were experiencing the same or similar problems.

Of the LAPHTs who participated, some DsPH did not have time to be interviewed and suggested a non-DsPH colleague. A few of these were unfamiliar with central government communication lines during the pandemic. This was a small number, and we believe the impact on the overall data quality was minimal.

### 4.2 Impacts on research and policy

The key lessons from LAPHT experiences were the importance of strong relationships with NHS staff, and community engagement. How these can be developed and maintained in the longer term without future investment was a concern. Stronger relationships and partnership working between public health and the NHS should ensure clarity about the roles and responsibilities of each.

The wider results reinforce and enhance the findings from previous research demonstrating the importance of incorporating local public health infrastructure, expertise and existing relationships into national vaccination planning. A policy brief with our recommendations has been shared with the UKs Department of Health and Social Care [26].

Future research should capitalise on public health, NHS staff and community relationships, and explore the design and implementation of PH/NHS joint service delivery models to tackle health inequalities. These delivery models should be informed by experiences of the Covid-19 vaccination programme and with input from community partners.

What this study adds

• The views and experiences of DsPH and local authority staff during the turmoil of the Covid pandemic and the roll out of the vaccination programme
• Reinforces and builds upon previous research findings demonstrating the importance of incorporating local public health infrastructure, expertise and existing relationships into national vaccination planning
• With the transition of public health into local authority, NHS staff understanding of their role, knowledge and expertise appears to have been lost
• The importance of developing and maintaining relationships with NHS staff

Implications for Policy and Practice.

• Future research should explore the design and implementation of PH/NHS joint service delivery models to tackle health inequalities
• New models of delivering care to disadvantaged populations should be informed by the experiences of the Covid-19 vaccination programme and with input from community partners

## Declaration of competing interest

The authors declare that they have no known competing financial interests or personal relationships that could have appeared to influence the work reported in this paper.

## Footnotes

1 Primary Care Networks were groups of general practices who work together to provide integrated services.
