# Supplementary material for: Reaching national Covid-19 vaccination targets whilst decreasing inequalities in vaccine uptake: Public health teams' challenges in supporting disadvantaged populations
Source: Public Health Pract (Oxf). 2024 Oct 25;8:100551. doi: 10.1016/j.puhip.2024.100551 (PMC11564988; doi:10.1016/j.puhip.2024.100551)
Supplement: Multimedia component 1 [file mmc1.docx]

Addressing local and ethnic inequalities in Covid-19 vaccine uptake: a place-based approach

**TOPIC GUIDE FOR INTERVIEWS**

- **Individual/team and population information**
- Confirm role
- Length of time in post
- Number in team/number devoted to vaccine programme/initiatives
- ?Population size of LA
- % in disadvantaged groups
- **Initiatives**
- List and briefly describe initiative(s)
- How was the choice of initiative(s) informed?
- Target for initiatives and how identified
- How is the intervention documented?
- Is there a formal evaluation?
- What data sources are available?
- **Experiences of delivering initiatives**
- What has your local team been doing *within* these initiatives?
  - PROBE - Have they changed or altered anything about the initiative? If so, why?
- How have these initiatives been received by the groups targeted?
- What has worked/not worked?
- What has been learned?
- Would you do anything differently in future similar situations? If so, why, if not, why?
- How were national policies perceived in relation to the initiatives?
  - PROBE did they help or hinder?
- **Future**
- Will you implement the same initiatives for the booster programme?
- Is there anything you have learned you would apply or carry forward into other public health interventions?
