# Supplementary material for: Reaching national Covid-19 vaccination targets whilst decreasing inequalities in vaccine uptake: Public health teams' challenges in supporting disadvantaged populations
Source: Public Health Pract (Oxf). 2024 Oct 25;8:100551. doi: 10.1016/j.puhip.2024.100551 (PMC11564988; doi:10.1016/j.puhip.2024.100551)
Supplement: Multimedia component 2 [file mmc2.docx]

PHIVU

Codes

| Overarching code | Sub-codes |  |
| --- | --- | --- |
| BOTTOM UP APPROACH |  |  |
| Importance of work with communities |  |  |
| Pre-Covid and pre vaccine work with communities |  |  |
| CENTRAL GOVERNMENT |  |  |
| Attitude to/understanding of local govt and local context | Acknowledging experience and skills of local authority PH staff  DsPH should be part of the solution  Harnessing intelligence from communities |  |
| Command and control |  |  |
| Covid messages |  |  |
| Funding | Per vaccine funding perpetuates inequalities |  |
| NHS | Getting NHS to listen/time taken to convince NHS colleagues (to try initiatives)  How NHS operate  Liberating staff to support vaccine initiatives  Success has surprised NHS |  |
|  |  |  |
| National policy and communication with DsPH | Ever changing policy  Policy should encourage partnership working  Plans not always thought through or complete  Restrictions on DPH communication with public  Regular press briefings should have continued  Short time for PH teams to respond to changes  Not quick to myth bust |  |
| Restrictions on data |  |  |
| Inequalities | Tackling inequalities should be on all agendas  Understand inequalities and poverty  Understanding diverse communities  Not always about targets and number vaccinated |  |
| Support from DHSC and NHS England |  |  |
| CHALLENGES |  |  |
| Access to resources |  |  |
| Antivax activities and impact |  |  |
| Changes to public health |  |  |
| Cohorts |  |  |
| Communicating within boundaries set by government |  |  |
| Constant change |  |  |
| Counteracting low level of trust in government and authority |  |  |
| Data |  |  |
| Different priorities NHS and PH | Offering the vaccine is not enough  Having to provide evidence to NHS for target areas  Getting buy in from NHS colleagues  Maintaining momentum with NHS partners  Low critical mass, costs and waste  PCNs dropping out of vaccination programme  Staffing popups from PCNs |  |
| Vaccine programme | Fragmented  Mass vaccination sites  National booking system  Speed of vaccination programme  Vaccinating children  Younger cohorts |  |
| Funding |  |  |
| Link into communities | Communities difficult to reach  Reaching the unvaccinated |  |
| Misinformation |  |  |
| Responding to concerns raised in the media |  |  |
| DETAILS OF AREA |  |  |
| Geography |  |  |
| Layout |  |  |
| Make up of region and proportions of disadvantages groups |  |  |
| INITIATIVES |  |  |
| Importance of understanding the area |  |  |
| Longstanding relationships with communities | Trust in community partners |  |
| NHS initiatives |  |  |
| Providing venues for vaccination | Vaccine sites and hubs |  |
| Initiatives to tackle vaccine inequalities | Description  Initiatives that haven't worked so well |  |
| Work undertaken to support initiatives |  |  |
| INTERVIEWEE PROFILE AND TEAM INFORMATION |  |  |
| Experience of pandemics |  |  |
| Profile of interviewee |  |  |
| Role of PH in vaccine programme |  |  |
| Team involved in vaccine work |  |  |
| LESSONS |  |  |
| Building relationships with vaccine providers | Listening to NHS partners  Praise for NHS  Working with NHS |  |
| Building upon and maintaining contacts and relationships | Relationships that have developed |  |
| Importance of community capacity building |  |  |
| Learning for future initiatives re health inequalities | Problems would like to address |  |
| PUBLIC HEALTH AND NHS RELATIONSHIPS |  |  |
| Covid raised awareness of other orgs |  |  |
| Experience of working with NHS pre covid |  |  |
| NHS relationships and ways of working |  |  |
| SHARING AND LEARNING |  |  |
| Comparison with other local authority areas |  |  |
| Learning from what has gone before |  |  |
| Sharing with and learning from other LAs |  |  |
| Work with other LA |  |  |
| STRUCTURES |  |  |
| Lines of communication with govt DHSC etc |  |  |
| Local authority information and structure |  |  |
| Local structures DsPH |  |  |
| Structure of inequalities vaccine work |  |  |
| UNDERSTANDING VACCINE UPTAKE AND INEQUALITIES |  |  |
| General comments about inequalities |  |  |
| Research undertaken locally |  |  |
| WHERE TO BEGIN AND MONITORING UPTAKE |  |  |
| Deciding upon initiatives | Knowledge of population  Understanding the issues  Who are the disadvantaged groups and unvaccinated |  |
| Ongoing monitoring of uptake |  |  |
| Ways of identifying target groups | Data from other vaccination programmes |  |
| What to do if initiative is not working |  |  |
