# Supplementary material for: Reaching national Covid-19 vaccination targets whilst decreasing inequalities in vaccine uptake: Public health teams' challenges in supporting disadvantaged populations
Source: Public Health Pract (Oxf). 2024 Oct 25;8:100551. doi: 10.1016/j.puhip.2024.100551 (PMC11564988; doi:10.1016/j.puhip.2024.100551)
Supplement: Multimedia component 3 [file mmc3.docx]

Table 5– Initiatives to improve access to Covid vaccination

| Initiatives | Number of LA’s implemented | Quotations |
| --- | --- | --- |
| Workplace vaccination | 7 | *‘One thing we did terms of rural communities, we have mushroom farm and a pig farm with a lot of migrant workers, some of them potentially undocumented migrant workers and we worked with the primary care network to deliver a vaccine clinic on that site.‘* Site 8 September 2021 |
| Transport to mass vaccination sites | 6 | ‘W*e’ve got a link with the port authority so when the ships come into [dock] they’re obviously coming from international waters and different countries. So we’ve been able to transport them to vaccination sites to get vaccinated.’* Site 9 October 2021 |
| Vaccination hubs (shops, community centres, pharmacies) | 8 | ‘W*e’ve got a range of local vaccination hubs that have been operating more or less since day one. […] One is right in the heart of (place) and that’s a really helpful location, that one in particular because of the large BAME population who live around there.’* Site 11 December 2021 |
| Outreach/targeted clinics for specific communities (homeless, mosques, housebound, deprived, deaf communities) | 14 | *‘Our testing outreach team was also our covid vaccine promotion team and they’ll be in different places – like they’ll be in the shopping centre in (place) which is a much more downbeat town centre So, they’ll be in places where people from more deprived backgrounds are more likely to be, and where our ethnically diverse populations are, like outside Morrisons for example and that kind of thing.’* Site 7 September 2021 |
| Mobile vaccine units, drop-ins and pop-ups | 21 | *The vaccination bus has been used for workplaces, and we’ve also taken it to community locations, for example in the city of (name) where we’ve had quite a low uptake of the vaccination, so we’ve plonked it in places where we know there’s going to be this footfall going past it and picked up probably a few hundred in total vaccinations as a result of that over the days we did that.’* Site 1 July 2021 |
