# Supplementary material for: Reaching national Covid-19 vaccination targets whilst decreasing inequalities in vaccine uptake: Public health teams' challenges in supporting disadvantaged populations
Source: Public Health Pract (Oxf). 2024 Oct 25;8:100551. doi: 10.1016/j.puhip.2024.100551 (PMC11564988; doi:10.1016/j.puhip.2024.100551)
Supplement: Multimedia component 4 [file mmc4.docx]

Table 6 – Initiatives to improve confidence in, and complacency about, Covid vaccination

| Initiatives | Number of LA’s implemented | Quotations |
| --- | --- | --- |
| Covid Community Champions/community partners | 20 | *'They were recruited and trained and supported to have messages at their fingertips around covid and pointing people towards the latest guidance and we wanted people who were able to have conversations at the school gate or conversations at the local social club and that sort of thing and then we recruited 60 or 70 covid champions.’* Site 11 December 2021 |
| Use of trusted voices in messaging | 11 | *‘We’ve got a number of Asian GPs who have been fantastic advocates for us to say, ‘No I’m telling you, you should be doing this,’ and working with the mosques and working with some of the infrastructure that goes with some of the minority groups.’* Site 17 January 2022 |
| Street teams (door to door engagement) | 9 | *‘We paid for people to door knock to say, “Can we talk to you about vaccination?” not saying that they should or shouldn’t just letting them know and quite a lot of the time we found that one of three things: one, they were waiting; two, they didn’t know and if they could do it now, if they could do it; and three, no they weren’t going to.’* Site 12 December 2021 |
| Promoting vaccination through on-line advertising | 6 | *‘Our coms team and the CCG coms team worked together to pull together information that got translated into five different eastern European languages and pushed that out through paid Facebook advertising to anyone who has been in the (name) area and Facebook can identify if your profile is predominantly in another language so they can push the adverts in that language to that profile.’* Site 3 August 2021 |
| Videos/podcasts PH Teams/community group members | 11 | *‘We found the most interesting people came forward, we communities came and said, ‘what can we do?’ a lot of the community leaders said, ‘I’ll make a video for you, just tell me the…’ you know, ‘I’ll make a video for you, and I’ll put it out to all my networks.’* Site 7 September 2021 |
| Targeted communications | 7 | *‘The comms messaging has been developed for different subgroups of the population. For younger people we realised that there was quite a bit of misinformation, myths that needed to be addressed so we’ve targeted comms around addressing the myths that young people or younger people have been listening to on social media etcetera so that was one.’ Site 13 October 2021* |
| Support groups and sessions (Q&A) | 6 | *‘We’ve had what I’d call super-targeted and that’s been with our NHS professionals and our care home professionals, and we’ve had a working group, since they’ve really become eligible focusing on care homes with less than 50% uptake doing additional Q &A specifically for their staff.’* Site 4 August 2021 |
| Leaflets | 9 | *‘We had teams of staff going up and down streets leaflet dropping in particular streets where we know there’s low uptake. Those sort of hyper local type interventions, I think are probably really, really useful going forward with wider inequalities agendas.’* Site 1 July 2021 |
| Engagement through Faith groups | 8 | *‘We’ve also got a faith forum that all of the different religions and religious leaders were part of. We attended their meetings at the mosque and ran specific sessions for the Imams for the community as well. […] I think it worked well actually. We did have a chance to share as much information as possible, enable people to talk to us and different people, and enabling people to have one to one conversations, and ask questions.’* Site 19 February 2022 |
| Helping people to talk confidently about Covid and vaccine | 4 | *‘Who is it who engages with the people who have lower vaccine uptake, who engages with the residents on the lowest incomes for example? So, housing officers, for example, social housing, we’ll train those people, people who work in customer services at the council benefits, housing needs, we train them motivational interviewing, so how do you answer base questions about the vaccines, signpost people but how do you also have conversations with somebody who is reluctant to get vaccinated in a way that doesn’t massively get their back up?’ Site 6 September 2021* |
| Direct telephone contact | 2 | *‘We had a team whose normal job is social prescribing link workers, they’re employed by the council but work in GP practices so have all the sort of information governance stuff ticked off and they were able to sit in the practices and go through lists of patients who were eligible and hadn’t been vaccinated yet and phone them up and speak to them.’ Site 5 September 2021* |
